# Tribe Paniceae Cereals with Different Ploidy Levels: *Setaria italica*, *Panicum miliaceum*, and *Echinochloa esculenta*

**DOI:** 10.3390/genes16040426

**Published:** 2025-04-01

**Authors:** Kazuhiro Satomura

**Affiliations:** Department of BioScience, Nagahama Institute of Bio-Science and Technology, Shiga 526-0829, Japan; k.satomura@nagahama-i-bio.ac.jp

**Keywords:** Poaceae, whole genome duplication, polyploidy, polymorphism, evolution

## Abstract

Plants have repeatedly undergone whole-genome duplication during their evolutionary history. Even in modern plants, there is diversity in ploidy within and between species, providing a snapshot of the evolutionary turnover of ploidy. Here, I will review the diversity of ploidy and the evolution of the genome constitution, focusing on the millet species *Setaria italica*, *Panicum miliaceum*, and *Echinochloa esculenta*. These are all historically important cereal crops that have been domesticated in East Asia. They all display a basic chromosome set of nine, but they are diploid, tetraploid, and hexaploid, respectively. The timing of ploidy is different among the millet species, as is the extent of gene family expansion and gene loss. There also exists complex subgenomic evolution in the wild species within each genus. These three millet species and their related wild species are suitable models for elucidating the molecular evolution and diversity of genome duplication by comparative genomic analysis.

## 1. Introduction

Foxtail millet (*Setaria italica*), proso millet (*Panicum miliaceum*), and Japanese barnyard millet (*Echinochloa esculenta*) are historically important cereals that were domesticated in East Asia [1]. All three of these millets are grasses of the tribe Paniceae, subfamily Panicoideae, family Poaceae. These three millets are C_4_ plants, which suggests that they are highly efficient in their use of water and nitrogen, and can tolerate high temperatures, drought, saline soils, and poor nutrition [2]. In addition, these millets are also able to adapt to colder regions than can rice, so in ancient times, these were widely cultivated in high-latitude regions where rice could not grow [1,3]. Today, these millets are the focus of global attention as food, fodder and forage, and biofuels.

These three millet genomes display different cytotypes, and their basic chromosome number is nine: *S. italica* is diploid (2n = 2x = 18), *P. miliaceum* is allotetraploid (2n = 4x = 36), and *E. esculenta* is allohexaploid (2n = 6x = 54). Polyploid is a cytotype expressing an additional complete set of chromosomes, and each set of chromosomes in a polyploid organism is called a subgenome. Polyploidy is thought to result from errors in mitosis or meiosis or from hybridization between individuals with different genomes [4,5]. Polyploidy is common in plants and varies within and between species [6,7]. The number of species with multiple cytotypes in the angiosperms is uneven among families [8]. In addition, polyploid species are more common at higher latitudes, which is thought to be related to temperature [9]. This geographical cline is also common in animals [10]. Polyploidy provides genetic diversity and redundant genes, and in evolution, it can enhance environmental adaptation and the acquisition of new gene functions [11]. Polyploidy is thought to be one of the factors affecting plant speciation because it can create isolation barriers [12,13]. However, gene flow between polyploids, especially gene flow to high levels of polyploidy, is common in nature, so the degree of isolation and gene flow must be carefully discussed [14]. In addition to these characteristics, polyploids increase biomass, so there are many examples of polyploids in cultivated plants in the history of domestication and in modern breeding [15,16,17,18]. For example, fonio millet (*Digitaria exillis*) is an allotetraploid crop that was domesticated from the wild allotetraploid grass *Digitaria longiflora* [19]. The domestication history of allohexaploid wheat *Triticum aestivum* is more complex. Emmer wheat was created by the domestication of the allotetraploid *Triticum turgidum* subsp. *dicoccum* from the wild allotetraploid *Triticum turgidum* ssp. *dicoccoides* and then further hybridization with diploid Tausch’s goatgrass *Aegilops tauschii* (*Aegilops squarrosa*), producing allohexaploid wheat [20,21,22,23]. The wild ancestor of *S. italica* is a diploid weed *Setaria viridis* [24], and the wild ancestor of *E. esculenta* is an allohexaploid weed *Echinochloa crus-galli* [25]. On the other hand, it is still unclear what evolutionary history led to the domestication of *P. miliaceum* from the wild genus *Panicum*. The reference genome of a cultivar Yugu1 strain of *S. italica* was released in 2012 [26], and a wild strain A10.1 of *S. viridis* was released in 2020 [27]. The first nearly complete genome assembly for an elite strain Longmi4 [28] and a cultivar [29] of *P. miliaceum* was released in 2019, and the chromosome-level reference genome of a cultivar AJ8 of *P. miliaceum* was released in 2024 [30]. The scaffold-level reference genome of a wild strain STB08 of *E. crus-galli* was released in 2017 [31]. The genome size of *S. italica* is small at 400 Mb, and it has been studied as a model species for millets. In recent years, the infrastructure for genomic research of the tribe Paniceae has been established.

The angiosperm model *Arabidopsis thaliana* is a diploid plant and has a small genome of 125 Mb [32]. However, the multiple nested segmental duplications in the genome suggest that it has experienced two whole-genome duplication (WGD) events (α and β) and one triplication event (γ), along with repeated diploidization with gene loss during its evolution [32,33,34,35]. Furthermore, a comparison of the synonymous substitution rates between paralogs suggests that WGD also occurred in the lineages of the common ancestor of flowering plants (ε) and the common ancestor of seed plants (ζ) [36,37]. Such ancient polyploidization events are called paleopolyploidization. Paleopolyploidization has occurred independently in different lineages many times in the evolutionary history of plants [38]. Paleopolyploidization events in various plant lineages are thought to have occurred independently around the Cretaceous–Paleogene (K-Pg) boundary, so it is thought that this event helped plants to survive the mass extinction event by adapting to their drastically changed environment [39,40,41]. The loss of a duplicated gene usually occurs in a short period of time, and the average half-life is estimated to be around 4 million years [42]. In plants, however, it is estimated to be around 20 million years [38]. The WGD and the gradual gene loss promote plant radiation and enhance the acquisition of new plant traits. There are many gene and domain families that have converged back to singletons after independent genome duplications in different eukaryotic taxa [43]. On the other hand, there are cases where the number of genes has increased due to WGD and gene duplication, such as the MADS box transcription factor gene family in plants, resulting in the acquisition of sub-functions or new functions of genes and diversity in the gene set between species [44]. It has been proposed that gene duplication in molecular evolution provides redundancy in genes and drives their functional evolution [45]. Plants are an important model for studying the evolutionary significance of genome and gene duplication, as these are often observed in plants. What is the degree of diversity in polyploidy and gene duplication in plants? And how does it affect the molecular and adaptive evolution of wild and domesticated species? Here, I will summarize the diversity and evolution of polyploids and gene families by focusing on the three millet species *S. italica*, *P. miliaceum*, and *E. esculenta*.

## 2. Ploidy in Cereals

**Figure 1:**
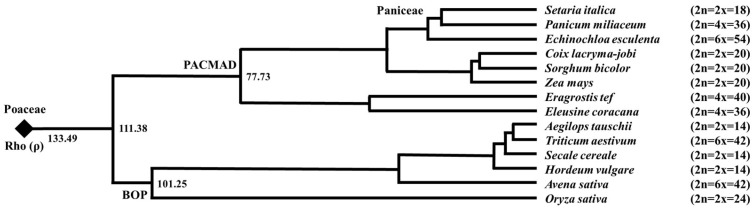
Phylogenetic relationships and estimated divergence times (million years ago) of 14 cereals of Poaceae, modified from Zhang et al. [52]. The black diamonds indicate the common Poaceae WGD event rho (ρ). In addition to a whole genome duplication, gene family expansions due to, e.g., tandem and segmental duplications, have occurred repeatedly in the Poaceae. It has also been suggested that there have been several lineage-specific WGD events. Some cereals have experienced polyploidization before and after domestication, and the current cytotype is indicated in parentheses to the left of the species name.

The most recent common ancestor (MRCA) of cereal crops experienced either two WGDs (ζ and ε) [37] or only one WGD (ζ) [46], and these are shared among all flowering plants. Subsequently, there were τ, σ, and ρ WGD events before cereal radiation [47,48,49,50]. After the rho (ρ) event, the common ancestor of Poaceae displayed a cytotype of 2n = 24 due to WGD and chromosomal duplication and fusion [51]. The current grass cytotype was formed by subsequent chromosomal fusion and the insertion of chromosomes into the centromere region of other chromosomes (Figure 1).

During the process of grass diversification, there were also numerous shared and lineage-specific WGDs [52]. Most of the WGDs that have been reported after the rho (ρ) in Poaceae are recent events, such as those within the genus. For example, as described above, wheat became a hexaploid through complex hybridization after domestication [20,21,22,23]. There is a diversity of ploidy within the genus *Avena*, and oats (*Avena sativa*) were domesticated from the hexaploid wild species *Avena sterilis* [53]. Similarly, teff (*Eragrostis tef*) and finger millet (*Eleusine coracana*) were also domesticated from tetraploid wild species [54,55]. Plants sometimes undergo polyploidization before and after domestication. The polyploidization of *P. miliaceum* and *E. esculenta* illustrate such cases.

## 3. Ploidy and Domestication History in Genus *Setaria*

The most reliable place of origin of *S. italica* is the Yellow River Basin in northeastern China. The oldest archaeological evidence of *S. italica* in the world comes from grains found in excavated storage pits at the Cishan site about 8000 years ago [56]. In early times, its grain size was close to that of wild species, and isotopic data showed that millet constituted less than half of the staple diet. The ancient civilization was still a hunter-gatherer society and shifted to an agricultural society about 7000 years ago [57]. Since then, it has been suggested that millet has provided a sufficient yield of food for humans and feed for livestock diets in northeastern China for over 2000 years [58]. Around 5500 years ago, migrations of agricultural populations led to the spread of millet cultivation areas throughout Eurasia [59,60]. Since domesticated species gradually lose genetic diversity as they spread to neighboring regions, the population at the place of origin of domestication is expected to display the highest genetic diversity. The diversity center of *S. italica* is located in northeastern China, so the results of the population genome analysis also suggest that this is its place of origin [61].

The ancestral wild species of *S. italica* was identified as *S. viridis*, based on morphological and cytological evidence, as well as random amplified polymorphic DNA (RAPD) analyses, amplified fragment length polymorphism (AFLP) analyses, and other genetic markers [24,62]. *S. viridis* is a self-compatible annual grass with a small plant size and various environmental adaptations. Environmental adaptation in *S. viridis* is attributed to its high phenotypic plasticity [63]. *S. italica* has lost about half of its genetic diversity during the history of its domestication, which is the same level as rice [64]. However, when considering that rice lost 70% of its genetic diversity during the domestication process, it can be said that the genetic diversity of *S. viridis* is not particularly high [65]. *Oriza rufipogon* and *O. nivara*, which are thought to be the wild ancestors of the domesticated rice, *O. sativa*, are distributed from India to Southeast Asia, and relative wild species of the genus *Oryza* are generally distributed in limited areas of the tropics [66]. Therefore, it is thought that *S. viridis* was also originally distributed in a limited area somewhere around northeastern China. However, *S. viridis* is now distributed in urban and rural areas from the tropics to the subarctic zone around the world, and it is thought that it has spread as a companion weed of *S. italica* or through human migration and interaction. *S. italica* and *S. viridis* share 75% of their genetic variation due to spontaneous interspecific gene flow, which occurs in both directions at rates ranging from 0.002% to 0.6%, depending on plant density and distance [64,67]. Domesticated species generally reduce their genetic diversity through bottlenecks and artificial selection, but *S. italica* could recover it through hybridization with the companion weed *S. viridis*. Identical cytotypes in domesticated and ancestral wild species can facilitate gene flow through hybridization.

Most species of the genus *Setaria*, with over 100 species recorded, are distributed from the tropics to the subarctic, growing wild on grasslands, farmlands, and roadsides. Most of *Setaria* species are self-compatible annual grasses, producing hundreds of dense seeds from a single inflorescence. According to the Chromosome Count Database (CCDB), about half of the species in the genus *Setaria* exhibit intraspecific cytotype polymorphisms [7,68]. In addition, the standard cytotype has a wide range of chromosome numbers within the genus, from 18 in diploid species to 72 in tetraploid species, with a maximum of 108 reported for the dodecaploid species *S. rosengurttii* from South America. Polyploidy is related to the size of the plant cells and organs [69,70], and it seems to be related to the size of the *Setaria* species, but since plant size is also affected by the environment, this should be clarified by research designed to distinguish between genetic and environmental factors. The set of subgenomes in polyploids may differ within and between species, so in the genus *Setaria*, the correspondence of subgenomes between species has been investigated using genomic in situ hybridization (GISH) or nuclear DNA markers [71,72,73,74,75,76]. Here, the correspondence between subgenomes according to GISH is summarized in Table 1. First, the diploid genome of *S. italica* was designated as genome A [24]. The genome structure also supports that *S. viridis* is the wild ancestor of *S. italica*. Next, a diploid *S. adhaerans,* with a genome distinct from genome A, was found and named genome B [73], and a diploid *S. grisebachii,* with a genome distinct from both genomes A and B, was found and named genome C, according to GISH [74]. After genome D, the classification was based on molecular phylogenetic analysis using the 5S rDNA sequence [76]. The GISH results indicate that many of the polyploids in the genus *Setaria* are recent, and only a few species display different subgenomes that diverged in ancient times through interspecific hybridization. However, previous studies have been confronted with the problem of confusion regarding species identification. It is difficult for researchers to keep track of all the species of the genus *Setaria* distributed around the world, so it is not easy to determine whether samples from one’s own country and samples from overseas studies are the same species. The genus *Setaria* often exhibits morphological polymorphisms that are genetic or environmental, making species identification even more difficult. Furthermore, since hybridization can also occur between closely related species, a high level of knowledge and skill is required to distinguish between them. For example, in Japan, there have been reports of a hybrid species of *S. viridis* and *S. italica* called *S. x pycnocoma*, which may have become gigantic as a result of the gene flow from *S. italica* into *S. viridis*, without knowledge concerning this species, it might be confused with *S. faberi*. The *S. leucopila* in Table 1 originates from Germany, and it is assumed to be a diploid of genome A [76], but samples from North America have been reported to be hexaploid to dodecaploid [77,78], so it seems necessary to confirm that these are really the identical species. *S. verticillata* was thought to exhibit intraspecific polymorphism with the diploid and tetraploid forms, but the results of GISH suggested that the diploid *S. verticillata* is the same species as *S. adhaerens* [74]. The tetraploid genome of *S. faberi* has been thought to be an AABB allotetraploid [73], but molecular phylogenetic analysis of the 5S rDNA sequence showed that the other subgenome is also closely related to genome A [76]. The confusion in previous studies regarding the evolution of genome composition in the genus *Setaria* is due to the difficulty in identifying species and the reliance on analysis using small regions of the genome. In fact, there can be multiple mutations, translocations, and parallel loss of paralogs between subgenomes during molecular evolution. Therefore, these problems will be solved in the future via genome-wide molecular phylogenetic analysis.

## 4. Ploidy and Domestication History in Genus *Panicum*

The most reliable origin of *P. miliaceum* is the Yellow River Basin in northeastern China, the same area of origin as *S. italica*, but the oldest archaeological evidence in the world, which is about 10,000 years old, was discovered in a storage pit at the Cishan site [56]. Large quantities of grains carbonized more than 7500 years ago have also been excavated from neighboring sites [58]. These areas are located inland, marked by low rainfall, strong winds, and cool temperatures, and lacking water and nutrients in the soil [79]. Among the cereals, *P. miliaceum* is characterized by the lowest water and nutrient requirements, and its short growth period contributes to its high value in the region. Although *P. miliaceum* and *S. italica* may have been domesticated at different times, the two millets were used in the same region and the same culture, and they spread across Eurasia at the same time, following similar routes [59,60]. Population genomic analyses of *P. miliaceum* also indicate that the diversity center is located in northeast China [80,81].

The main cultivated species of the genus *Panicum* currently grown for food are *P. miliaceum* and little millet (*P. sumatrense*). *P. sumatrense* is small but morphologically similar to *P. miliaceum* and is currently cultivated mainly in South and Southeast Asia. It is thought to have been domesticated from a wild species about 5000 years ago during the Indus civilization, and large quantities of its seeds have been excavated at Harappa [82,83]. The ancestral wild species of *P. sumatrense* is still unclear. One candidate species is *P. psilopodium*, which is morphologically similar to *P. sumatrense* and can interbreed with *P. sumatrense*. Therefore, *P. psilopodium* is the ancestor of *P. sumatrense* or the wild *P. sumatrense* that became feral in ancient times [84].

There is a missing link in the evolutionary history of *P. miliaceum*, which was domesticated from the wild *Panicum* species. However, there are two weed subspecies of *P. miliaceum*. One is *P. miliaceum* subsp. *ruderale*, which was first described in Manchuria in northeastern China and is very similar to cultivated millet, except for some morphological differences [85,86]. To date, weedy millet has been reported from a wide range of areas, including Eastern Europe, the Aral and Caspian Sea basins, Bukhara in Central Asia, Siberia, and North America. The other subspecies is *P. miliaceum* subsp. *agricolum*, which has intermediate characteristics between those of cultivated *P. miliaceum* and the weedy *P. miliaceum* subsp. *ruderale*, and it has been found in Europe and North America. It is thought to have feralized from cultivated *P. miliaceum*. Recent population genomic analysis has shown that the weed subspecies is related to the Chinese native species of *P. miliaceum* and is more ancestral when assessed by molecular phylogeny [81]. Based on the results of gene flow observations between cultivated and wild populations, *P. miliaceum* subsp. *ruderale* may be an ancestral wild species, with gene flow after domestication. However, the relationship between *P. miliaceum* and wild *Panicum* species is still unknown, and research is continuing, especially on the respective donors of the heterozygous tetraploid subgenomes.

**Figure 2:**
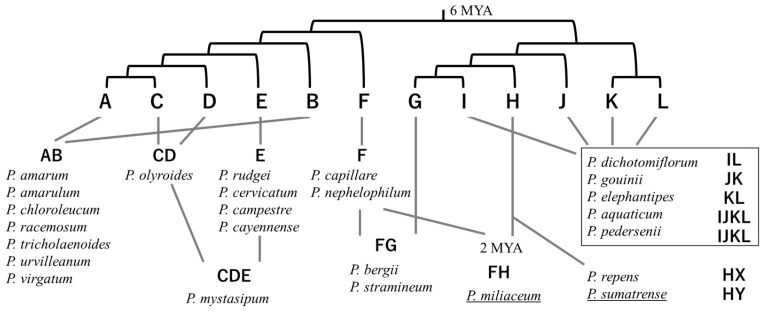
Summary of the phylogenetic relationships of subgenomes and species, as well as the chromosome composition of the genus *Panicum* [91]. Underlined species are domesticated species. X and Y represent unknown chromosomal types that differ from H [92]. Divergence times are based on comparisons of subgenomes of *P. miliaceum* [92,93].

Most of the approximately 450 species recorded in the genus *Panicum* are distributed from the tropics to the temperate zone and grow wild in grasslands, farmlands, wastelands, and coastal areas. The panicles have many small, sparsely attached seeds, and the larger panicles are over 60 cm long. According to the CCDB, about 30% of the species in the genus *Panicum* have been reported to display intraspecific cytotype polymorphisms, but there are also species such as torpedo grass (*P. repens*) and Guinea grass (*P. maximum*) that exhibit various ploidy polymorphisms that do not necessarily occur in multiples of nine [7,68]. *P. repens* is an allopolyploid species, and its irregular chromosome behavior during meiosis can sometimes result in the formation of abnormal pollen and gametes, reducing fertility [87]. *P. maximum* has also been reported to display abnormal meiosis, resulting in sterile pollen [88]. On the other hand, there are also *Panicum* species, such as *P. acuminatum* and *P. capillare*, which are always diploid, and *P. brevifolium*, which is always tetraploid, despite numerous reports to the contrary. There are also species that appear to have a chromosome number that is a multiple of 10, and the chromosome number within the genus *Panicum* is diverse [68]. The genus *Panicum* includes large, fast-growing species that can grow in nutrient-poor arid regions, thus attracting attention as a source of bioenergy. The whole genomes of Hall’s panicgrass (*P. hallii*) [89] and switchgrass (*P. virgatum)* [90] have been sequenced as models. Figure 2 shows the evolution of the complex genome constitution of the genus *Panicum*, according to the molecular phylogenetic relationships of subgenomes based on the sequences of five homologous nuclear genes [91,92,93]. Both domesticated species *P. miliaceum* and *P. sumatrense* exhibit allopolyploid genomes (2n = 4x = 36) [92]. The subgenomes of *P. miliaceum* are thought to have diverged about 6 million years ago and then become tetraploid by interspecific hybridization about 2 million years ago [28,92,93,94]. Based on GISH, the origin of the two subgenomes of *P. miliaceum* was suggested to be related to the diploid genome of witch grass (*P. capillare*) and one of the subgenomes of *P. repens*, respectively [92]. However, further research is needed to clarify how the subgenomes of *P. miliaceum* and *P. sumatrense* were derived by the hybridization of donor species in the complex evolution of the genome constitution of the genus *Panicum*.

The subgenomes of *P. miliaceum* are highly conserved, but it is suggested that four interchromosomal exchanges occurred during molecular evolution after tetraploidization [28,29]. About 75% of the gene families of *P. miliaceum* are shared among Poaceae species, and only about 4% are unique to the species [29]. The gene family that has expanded the most in *P. miliaceum* is the ubiquitin E3 ligase subunit, and the number of BTB domain sequences for the target recognition of E3 ligases in its genes is greater than that in other angiosperms, and it is thought that multiple duplications have occurred in the Paniceae lineage [29]. The genome of *P. miliaceum* displays many deleterious mutations caused by transposons, and more than half of the entire genome is composed of transposons [81,93]. There is biased pseudogenization occurring between subgenomes, and it is thought that the lost genes are compensated for by genes on the other subgenome [81,93,95].

## 5. Ploidy and Domestication History in Genus *Echinochloa*

Barnyard millet was used, along with rice, in the Yangtze River Basin in China around 10,000 years ago, but it does not appear that it was domesticated [96]. Even in the wild, barnyard millet has a short life cycle and can produce large grain sizes comparable to those of cultivated millets, so it is possible that collecting wild barnyard millet was sufficient at the time. The oldest carbonized seeds of wild barnyard millet (*E. crus-galli*) have been excavated in northern Japan from around 9000 years ago, and the continuous discovery of plant opals and plant remains from around 6000 years ago suggests that Japanese barnyard millet (*E. esculenta*) cultivation had already begun in Japan by that time [97,98,99]. Later, with climate change, the phenotype of Japanese barnyard millet grains changed, and stable excavations of large-sized grains similar to those of today have been found at archaeological sites in Japan dating from around 1000 years ago. Japanese barnyard millet is more resistant to cold damage than is rice, and until modern times, it was considered important in mountainous areas of Japan, northern Japan, and the Primorsky Krai [1].

The major cultivated species of the genus *Echinochloa* currently grown for food are *E. esculenta* and Indian barnyard millet (*E. frumentacea*). *E. frumentacea* is a domesticated cereal that has historically been cultivated in South Asia and Sub-Saharan Africa. Archaeological evidence suggests that cultivation began in India about 7000 years ago and in China about 5000 years ago [100]. *E. colona* overlaps its distribution area with the original domestication site of *E. frumentacea*, and based on their cytotypes and molecular phylogenetic relationships, it is thought to be the wild ancestor of *E. frumentacea* [25,31]. Both *E. esculenta* and *E. frumentacea* show intraspecific cytotypic polymorphism, and various strains with chromosome numbers ranging from 36 to 72 have been reported. *E. colona* exhibits a hexaploid genome structure like that of *E. crus-galli*, but the two subgenomes are of a different type than those of *E. crus-galli*, indicating that they have evolved differently from *E. crus-galli*.

**Figure 3:**
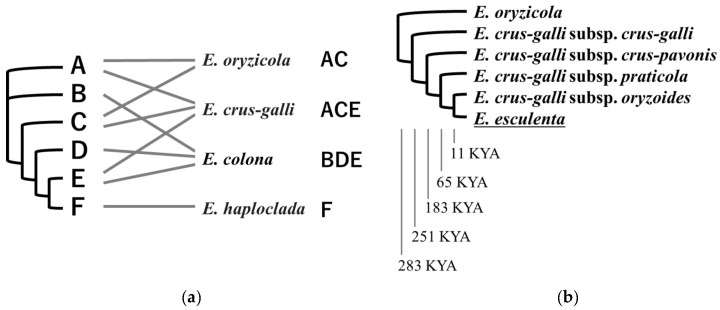
Phylogenetic relationships of *E. esculenta* and its wild relative species based on nuclear genome sequences, modified from the work of Wu et al. [102]. (**a**) Evolution of the subgenomes (A–F) of the genus *Esculenta* and their correspondence to species. (**b**) The divergence time (kilo years ago: KYA) of *E. esculenta* and *E. crus-galli* subspecies. Underlined species are domesticated species.

Most species of the genus *Echinochloa*, of which more than 50 species have been recorded, are distributed from the tropics to the subarctic zone and grow wild in wetlands and humid areas. The high phenotypic plasticity of the appearance of *Echinochloa* and morphological similarities across the genus have led to confusion in regards to taxonomy and species identification [101]. *Echinochloa* species are annual or perennial grasses that grow from 10 to 460 cm. Weeds of the genus *Echinochloa* generally grow faster and larger than rice in paddy fields, and in particular, *E. crus-galli*, *E. colona*, and *E. oryzicola* (also called *E. phyllopogon*) have spread worldwide as the most serious weeds in paddy fields. According to the CCDB, the basic chromosome number for the genus *Echinochloa* is x = 9, but many species are tetraploid or hexaploid, and the largest reported species is *E. polystachya*, which is a dodecaploid [7,68]. *E. esculenta* exhibits a set of three recently diverged subgenomes, and it is thought to have a complex history of speciation and subspecies diversification due to hybridization within and between populations (Figure 3) [102]. As a result of the frequent gene flow between *E. esculenta* and its wild relatives, the degree of domestication is thought to be less than that of other cultivated plants [102,103].

## 6. The Influence of Ploidy on Domestication and Selective Breeding

Paleopolyploidization has influenced plant evolution and is thought to be a factor in species diversification [12,13]. On the other hand, recently formed polyploid species display a higher extinction rate than that of their diploid relatives, so polyploidization is not always adaptive [104]. However, domesticated plants have experienced more polyploidization events than have wild species, and this is remarkable in monocotyledons [105]. There are several known pathways for the polyploidization of domesticated species [18], and post-domestication polyploidization can pose a barrier to isolation between domesticated and wild species [12,13]. The isolation barrier between domesticated and wild species may promote the fixation of artificially selected useful traits, but it will become more difficult to recover genetic diversity after the occurrence of bottlenecks caused by artificial selection or migration. However, even wheat, which underwent complex polyploidization after domestication, is known to have experienced gene flow from different polyploid strains, including wild species, during its history [106,107,108]. Different ploidy levels were found to be insufficient isolation barriers [14]. However, it is also possible that the rare hybridization with wild strains has had a beneficial effect on domesticated strains that have lost their genetic diversity, and so the effects of isolation due to ploidy and gene flow need to be carefully discussed.

Self-fertilization can also contribute to reproductive isolation, and it is often observed in domesticated species [109]. In Solanaceae, self-compatibility is known to be associated with polyploidy [110]. In the S-RNase system found in many flowering plants, including Solanaceae, there is one S-RNase that inhibits pollen tube elongation, but tandemly duplicated *S*-locus *F-box* genes (*SLF*s) can detoxify the non-self S-RNase [111]. In this system, if the *S* locus is duplicated, the duplicated *SLF*s detoxify their respective *S*-RNases, resulting in the emergence of self-compatible strains. Polyploidy can create isolation barriers due to cytotype mismatches or the development of self-compatible strains. However, although all three millets are self-compatible, they often have companion weeds with the same cytotype as their wild ancestral species or feralized strains. Thus, all three millets are cereals that have been domesticated in environments where they can acquire wild genetic diversity through gene flow. To genetically improve the three millets in the future, it may be better to genetically introduce a reproductive isolation mechanism into elite strains to prevent artificially modified genes from escaping into the wild.

Polyploidization provides opportunities for one of the duplicated genes to subfunctionalize, neofunctionalize, or pseudogenize or to acquire diversity in expression by creating genetic redundancy in molecular evolution [45,112,113,114]. For example, the glycoside hydrolase 3 (*GH3*) gene family, which is involved in hormone homeostasis and signaling pathways, is duplicated and functionally differentiated in Poaceae, and its copy number is higher in *S. italica* than in other crops [115]. On the other hand, in the glycosyltransferase family 8 (*GT8*), which is involved in the formation of cell walls, the gene family has diversified into seven subfamilies in the Poaceae, and *S. italica* has lost the GATL-related (GATR) subfamily [116]. The MADS box transcription factor genes involved in the establishment of floral organs have increased in gene family size by gene duplication in various lineages of angiosperms, and the duplicated genes have been retained, lost, or neofunctionalized [44,117]. A MADS box gene in *S. italica*, *SiMADS*, is involved in stress, such as drought, tolerance [118,119]. In the genus *Setaria* and *Panicum*, the MADS box gene family expanded mainly through segmental duplication, and cis-elements related to stress and hormone responses were conserved in the promoter region, but the size of the gene family tended to decrease [120]. Polyploidy is thought to be beneficial for adaptation to environmental stress [11], as it is associated with periods of historically drastic environmental change [39,40,41], and this may be related to stress response gene families that increase gene family size and functional differentiation. Plant resistance (*R*) genes are a family of genes that defend against pathogens and are also important in plant breeding [121]. This gene family has expanded and diversified through segmental and tandem duplications. It has been suggested that the leucine-rich repeat domains, which display pathogen recognition specificity, have been adaptively selected and have evolved and diversified through an arms race with pathogens [122]. More than 200 *R* genes with conserved functional domains were found in *S. italica*, many of which were tandemly repeated and showed syntenic similarity to other cereals [123]. In *S. italica*, it is suggested that the *R* gene singletons are under purifying selection, and that the tandem repeat genes have diversified and contributed to the acquisition of new functions [124]. There is much research on the evolution and function of duplicated genes in the millet *S. italica* model, but little in regards to *P. miliaceum* or *E. esculenta*. The pan-genome analyses of *S. italica* and *P. miliaceum* have revealed many genes involved in presence–absence variants (PAVs) caused by DNA transposons [61,81]. In *P. miliaceum*, PAVs included resistance genes and were biased between subgenomes [81]. Three millets are important as models for polyploidy and gene family evolution, and these comparative genomics studies will also provide useful genetic data for breeding.

Polyploids provide partial isolation barriers, and the increase in gene families is thought to have been important in the environmental adaptation of plants, therefore they were important in domestication. However, in breeding, gene deletion is sometimes necessary, but in polyploids, homologous genes must be deleted on all subgenomes. In East Asia, sticky grains of millet produced by the loss of the *waxy* gene are preferred for their flavor and cooking properties, while strains with the loss of the *waxy* gene were obtained in diploid *S. italica* and tetraploid *P. miliaceum* through historical unconscious selection [125,126]. Such strains were not produced in hexaploid *E. esculenta* until mutation breeding was established [1,127]. While diploids and polyploids have their advantages and disadvantages in regards to breeding, comparative genomic analysis of each will provide intrinsic insights into plant evolution and domestication.

## 7. Conclusions

The three millet species, *S. italica*, *P. miliaceum,* and *E. esculenta*, were domesticated in East Asia and have historically been important crops. These are easy to cultivate and resistant to environmental stress, so they have the potential to be cultivated even under the current climate changes, and they are expected to be a resource for food and biofuel in the future. While *S. italica* has been studied historically as a model for millets, the polyploids *P. miliaceum* and *E. esculenta* have been difficult to assemble genomically, and comparative genomic studies have not been possible until recently. The generalization of long-read sequencing is enriching genomic data, especially for cereals. Furthermore, with the development of comparative genomic analysis methods for polyploid plants, cereal genomes will provide insights into the evolution of various domesticated plants. However, comparative genomic analysis of wild polyploid plants is still poor because it requires genome data from model plants and many related species. Since cereals display a rich amount of data regarding their whole genomes and gene functions, comparative genomic analysis with their wild relatives would provide further insights. All three species belong to the tribe Paniceae, but they all express different ploidy levels. Therefore, the number of genes also differs significantly between species, which will affect environmental adaptation and molecular evolution. Furthermore, these three species display different ages of polyploidization, and *E. esculenta* is thought to have hybridized subgenomes that diverged within 0.3 MYA, and *P. miliaceum* has subgenomes that diverged around 6 MYA and hybridized subgenomes around 2 MYA, and the recent WGD in *S. italica* must be traced back to rho (ρ), which is a common event in the Poaceae. Therefore, the degree of progression to diploidization after polyploidization also differs between these species. Accumulating data on their wild relatives would help to clarify the effects of polyploidy on plant genome evolution.

## Figures and Tables

**Table 1:** Variations in sporophytic chromosome number, ploidy level, and genome constitution in genus *Setaria*.

Species	Number of Chromosomes	Median Ploidy Level	Genome
*S. italica*	18, 36	2n = 2x = 18	AA
*S. viridis*	18	2n = 2x = 18	AA
*S. leucopila*	18, 54, 68, 72, 108	2n = 2x = 18	AA?
*S. queenslandica*	18, 36	2n = 2x = 18	AA
*S. faberi*	36	2n = 4x = 36	AAAA? AABB?
*S. verticillata*	18, 27, 36, 54	2n = 4x = 36	AABB
*S. adhaerens*	18	2n = 2x = 18	BB
*S. grisebachii*	18	2n = 2x = 18	CC
*S. lachnea*	36	2n = 4x = 36	CCC’C’
*S. glauca*	36	2n = 4x = 36	DDDD
*S. parviflora*	36	2n = 4x = 36	DDDD
*S. plicata*	18, 36, 54, 72	2n = 4x = 36	EEEE?
*S. palmifolia*	34, 36, 54	2n = 6x = 54	EEEEEE?
*S. arenaria*	54	2n = 6x = 54	FFFFFF?

Question marks represent unknown or uncertain genome structures.

## Data Availability

No new data were created or analyzed in this study. Data sharing is not applicable to this article.
